# Impact of Community Treatment With Ivermectin for the Control of Scabies on the Prevalence of Antibodies to *Strongyloides stercoralis* in Children

**DOI:** 10.1093/cid/ciaa584

**Published:** 2020-05-18

**Authors:** Michael Marks, Sarah Gwyn, Hilary Toloka, Christian Kositz, James Asugeni, Rowena Asugeni, Jason Diau, John M Kaldor, Lucia Romani, Michelle Redman-MacLaren, David MacLaren, Anthony W Solomon, David C W Mabey, Andrew C Steer, Diana Martin

**Affiliations:** 1 Clinical Research Department, Faculty of Infectious and Tropical Diseases, London School of Hygiene and Tropical Medicine, London, United Kingdom; 2 Hospital for Tropical Diseases, University College London Hospitals NHS Trust, London, United Kingdom; 3 Division of Parasitic Diseases and Malaria, Centers for Disease Control and Prevention, Atlanta, Georgia, USA; 4 Atoifi Adventist Hospital, Atoifi, Malaita Province, Solomon Islands; 5 Kirby Institute, University of New South Wales, Sydney, Australia; 6 College of Medicine and Dentistry, James Cook University, Cairns, Australia; 7 Department of Pediatrics, University of Melbourne, Melbourne, Australia; 8 Department of General Medicine, Royal Children’s Hospital, Melbourne, Australia; 9 Tropical Diseases Research Group, Murdoch Children’s Research Institute, Melbourne, Australia

**Keywords:** scabies, neglected tropical diseases, ivermectin, Strongyloides

## Abstract

The prevalence of antibodies to *Strongyloides stercoralis* was measured in 0–12-year-olds using a bead-based immunoassay before and after ivermectin mass drug administration (MDA) for scabies in the Solomon Islands. Seroprevalence was 9.3% before and 5.1% after MDA (*P* = .019), demonstrating collateral benefits of ivermectin MDA in this setting.

*Strongyloides stercoralis* is unique among soil-transmitted helminths (STHs) in its ability to complete its life cycle within the human host. *S. stercoralis* infection is most commonly asymptomatic but may be associated with eosinophilia, fatigue, diarrhea, and occasionally larva currens [1]. People with compromised immune systems are at risk of potentially fatal hyperinfection syndrome [1]. Albendazole or mebendazole are effective against other major STH species and have been incorporated into mass drug administration (MDA) programs for public health purposes. However, these drugs have limited efficacy for strongyloidiasis, for which ivermectin is the first-line agent.

Ivermectin MDA is used widely to control a number of neglected tropical diseases (NTDs), including lymphatic filariasis, onchocerciasis, and most recently, scabies [2, 3], but has so far not been adopted for *S. stercoralis* control. Given the drug’s broad antiparasitic effect, ivermectin MDA may have as a collateral benefit the population-level control of *S. stercoralis*. Evaluation of *S. stercoralis* control has been facilitated by serological assays that detect antibodies against the NIE antigen, which is present in infective L3 larvae [4]. These antibodies likely indicate current or recent infection [5] and have high sensitivity and specificity compared with stool examination [4]. The NIE antigen has been adapted for use in the Luminex platform, allowing large-scale screening of populations using dried blood spots (DBSs) [6].

In the context of a community-randomized trial evaluating the addition of azithromycin to ivermectin-based MDA for scabies and impetigo in the Solomon Islands, we measured the prevalence of antibody responses to the *S. stercoralis* NIE antigen before and after MDA.

## METHODS

The trial of MDA for scabies and impetigo has been described elsewhere [7]. Briefly, selected communities in Malaita province in the Solomon Islands were randomized to MDA with open-label ivermectin or ivermectin plus azithromycin. All residents of these communities were eligible to participate. In both trial arms, all participants were examined for scabies and offered a single oral dose of ivermectin (200 μg/kg body weight). Persons with a contraindication to ivermectin (pregnancy, breastfeeding, or weight <15 kg) were offered topical permethrin instead. Those in whom a clinical diagnosis of scabies was made at baseline were given a second dose of ivermectin 7–14 days later. Written informed consent was obtained from adults and from a parent or guardian of each child aged under 18 years. Assent was also obtained from children who were able to provide it. The study was approved by the London School of Hygiene and Tropical Medicine Ethics Committee, the Solomon Islands National Health Ethics Committee, and the Atoifi Adventist Hospital Ethics Committee. The main trial was prospectively registered on clinicaltrials.gov (NCT02775617). Centers for Disease Control and Prevention (CDC) staff did not interact with study participants or have access to identifying information.

For the substudy reported here, we collected DBSs from all children aged less than 13 years at the baseline and 12-month surveys. We used a fluorescent bead-based assay to test for antibodies against the recombinant NIE antigen [6]. Briefly, serum was incubated with microspheres conjugated to NIE, beads were washed to remove unbound immunoglobulin (Ig), and then bound anti-NIE antibody was detected using biotinylated anti-human IgG + IgG4 antibody followed by streptavidin-phycoerythrin. Plates were run on a Luminex-200 (Austin, TX) and results reported as median fluorescence intensity with background subtracted (MFI-BG). We used a receiver operating characteristic curve analysis to determine cutoffs for seropositivity.

We conducted a before-and-after analysis to determine the effect of the MDA on the prevalence of antibodies to *S. stercoralis*. Azithromycin has no known activity against *S. stercoralis* so we combined the 2 trial arms into a single group for analysis. We calculated the seroprevalence of *S. stercoralis* at baseline and at 12 months and the absolute and relative reduction at 12 months. Statistical analysis was conducted in R 3.4.2 (R Foundation for Statistical Computing, Vienna, Austria).

## RESULTS

In total, 1291 people (including 553 children aged 0–12 years) were recruited and offered treatment for scabies at the time of the baseline survey. We collected DBSs from 539 of these children, representing more than 97% of children enrolled in the trial. At the 12-month follow-up, 1085 individuals (including 479 children aged 0–12 years) were seen, and we collected DBSs from 448 children (94%). At baseline, 9.3% of the children were seropositive for antibodies to NIE, with a range across the 6 study communities of 2.2% to 14.3% (Table 1). At the 12-month follow-up, the overall prevalence had declined to 5.1% with a range across the communities of 2.1% to 6.7%. The absolute difference in prevalence between baseline and 1 year was 4.2% (95% confidence interval [CI], .7–7.5%) and the relative reduction was 45% (*P* = .019). The seroprevalence of antibodies to NIE was lower at the follow-up visit than at baseline in all communities. Decreases were greater among children aged 5 years or older who would have received ivermectin, not permethrin, for treatment of scabies (Table 1). Neither baseline seroprevalence nor the magnitude of change following MDA was higher in communities with a higher baseline prevalence of scabies, where greater proportions of individuals would have received 2 doses of ivermectin.

**Table 1.** *Strongyloides stercoralis* Seroprevalence in Children Aged 0–12 Years Before and After Ivermectin Mass Drug Administration

	Baseline Seroprevalence, % (n/N)	12-Month Follow-up Seroprevalence, % (n/N)	Absolute Change, %	Relative Change, %
Age group				
Children aged 0–12 years (all participants)	9.3 (50/539)	5.1 (23/448)	−4.2*	−45.2*
Children aged 0–4 years	3.2 (9/279)	3.5 (8/225)	+0.3	+9
Children aged 5–12 years	15.8 (41/260)	6.7 (15/223)	−9.1**	−57.6**
Community				
1	14.3 (19/133)	5.2 (6/110)	−9.1	−36.4
2	2.2 (2/90)	2.1 (2/94)	−0.1	−4.5
3	11.4 (8/70)	6.9 (5/72)	−4.5	−60.5
4	9.0 (13/144)	6.7 (7/105)	−2.3	−25.6
5	7.8 (8/102)	4.9 (3/61)	−2.9	−37.2

*P < .05. **P < .01.

## DISCUSSION

Our study adds to the limited available data on the impact of ivermectin MDA on *S. stercoralis* prevalence. In the Northern Territory of Australia, a single round of ivermectin MDA reduced *S. stercoralis* seroprevalence from 21% at baseline to 5% at 6 months, and a second MDA at month 12 further reduced the seroprevalence to 2% at month 18 [8]. In Ecuador, the prevalence of *S. stercoralis* fell from 6.8% to zero following multiple rounds of MDA conducted for the purpose of onchocerciasis elimination [9]. Here, we showed that in 6 communities in Malaita, Solomon Islands, there was a decrease in seroprevalence of anti-NIE antibodies 1 year after a single ivermectin MDA conducted for the purpose of scabies control. The overall reduction in population seroprevalence was accompanied by decreases in the MFI-BG against NIE (Supplementary Table 1). The decrease was confined to those aged 5 years or older, who were much more likely to have received ivermectin, not permethrin, based on the weight cutoff of 15 kg. This strengthens the case that it was ivermectin MDA that caused the decrease.

There is no single gold-standard test for diagnosis of *S. stercoralis*. We used serological markers that correlate well with more direct measures of infection and are globally recognized as appropriate tests for both diagnosis and assessing post-treatment clearance. Had we combined serology with other tests such as Kato-Katz or polymerase chain reaction on stool specimens we may have detected more cases. We were, however, consistent in the measurement of exposure at both time points so are confident in the relative change observed. As complete sero-reversion of anti-NIE responses after treatment may take more than 1 year [5], and not all anti-NIE–positive individuals sero-revert after successful treatment [6], it is possible that we have underestimated the community-level effect of the intervention. Our study was not powered to detect changes in seroprevalence in each individual community; although community-level changes were not statistically significant, there were declines in seroprevalence in every community, consistent with our overall study finding. It is possible some individuals were reinfected between initial curative treatment at baseline and the 12-month survey. However even if reinfection did occur in some subjects, this does not negate the substantial overall population-level reduction seen.

Data on the prevalence and distribution of STHs in the Solomon Islands are limited. Two previous studies showed that hookworm, whipworm, and roundworm are common [10]. The baseline seroprevalence of *S. stercoralis* in children aged 0–12 years in the current study was 9.3%, which is broadly similar to the prevalence of other STH species reported in the Solomon Islands [10]. Current national deworming guidelines for the Solomon Islands are based on MDA of albendazole, which is likely to have little or no impact on *S. stercoralis*, and there is no routine access to diagnostics or treatment for *Strongyloides* nationally. Integrated strategies combining albendazole with ivermectin may therefore be beneficial for control of *S. stercoralis* as well as providing enhanced effectiveness against several STH species and allowing simultaneous control of scabies, which is highly endemic in the region [3, 7]. The current requirement for 2 doses of ivermectin, 1 week apart, for scabies MDA may represent a logistical barrier to integration as single-dose treatment is effective against *S. stercoralis* [11]. Newer antiparasitic drugs such as moxidectin show promise as single-dose treatment for scabies, strongyloidiasis, and other STHs [12, 13].

We have demonstrated that *S. stercoralis* is endemic in the Solomon Islands and added to the limited data demonstrating that ivermectin MDA, here conducted for the purpose of scabies control, can also reduce *S. stercoralis* seroprevalence. These data highlight ancillary benefits that can be conferred by NTD control programs and suggest that STH programs in this region may benefit from the addition of ivermectin alongside albendazole.

## Supplementary Data

Supplementary materials are available at *Clinical Infectious Diseases* online. Consisting of data provided by the authors to benefit the reader, the posted materials are not copyedited and are the sole responsibility of the authors, so questions or comments should be addressed to the corresponding author.

ciaa584_suppl_Supplementary_Data

ciaa584_suppl_Supplementary_Material

ciaa584_suppl_Supplementary_Table_1

## Notes

***Author contributions.*** M. M. wrote the first draft of the paper. S. G. and D. Martin conducted laboratory work. M. M., H. T., C. K., J. A., and R. A. conducted fieldwork. M. M., S. G., and D. Martin analyzed the data. M. M., J. D., J. M. K., L. R., M. R.-M., D. M., A. W. S., D. C. W. M., and A. C. S. designed and supervised the study. All authors revised the manuscript.

***Financial support.*** The study was supported by a Wellcome Trust, Clinical PhD Fellowship (grant number 102807 to M. M.). The US Centers for Disease Control and Prevention paid the laboratory costs.

***Potential conflicts of interest.*** The authors: No reported conflicts of interest. All authors have submitted the ICMJE Form for Disclosure of Potential Conflicts of Interest.
